# The main rhinovirus respiratory tract adhesion site (ICAM-1) is upregulated in smokers and patients with chronic airflow limitation (CAL)

**DOI:** 10.1186/s12931-016-0483-8

**Published:** 2017-01-05

**Authors:** Shakti Dhar Shukla, Malik Quasir Mahmood, Steven Weston, Roger Latham, Hans Konrad Muller, Sukhwinder Singh Sohal, Eugene Haydn Walters

**Affiliations:** 1 NHMRC Centre of Research Excellence for Chronic Respiratory Disease, School of Medicine, University of Tasmania, MS1, 17 Liverpool Street, Private Bag 23, Hobart, Tasmania 7000 Australia; 2 School of Health Sciences, University of Tasmania, Launceston, Tasmania 7248 Australia

**Keywords:** Intercellular adhesion molecule-1, Human rhinovirus, Epithelial adhesion, Chronic obstructive pulmonary disease, Chronic airflow limitation

## Abstract

**Background:**

ICAM-1 is a major receptor for ~60% of human rhinoviruses, and non-typeable *Haemophilus influenzae*, two major pathogens in COPD. Increased cell-surface expression of ICAM-1 in response to tobacco smoke exposure has been suggested. We have investigated epithelial ICAM-1 expression in both the large and small airways, and lung parenchyma in smoking-related chronic airflow limitation (CAL) patients.

**Methods:**

We evaluated epithelial ICAM-1 expression in resected lung tissue: 8 smokers with normal spirometry (NLFS); 29 CAL patients (10 small-airway disease; 9 COPD-smokers; 10 COPD ex-smokers); Controls (NC): 15 normal airway/lung tissues. Immunostaining with anti-ICAM-1 monoclonal antibody was quantified with computerized image analysis. The percent and type of cells expressing ICAM-1 in large and small airway epithelium and parenchyma were enumerated, plus percentage of epithelial goblet and submucosal glands positive for ICAM- 1.

**Results:**

A major increase in ICAM-1 expression in epithelial cells was found in both large (*p* < 0.006) and small airways (*p* < 0.004) of CAL subjects compared to NC, with NLFS being intermediate. In the CAL group, both basal and luminal areas stained heavily for ICAM-1, so did goblet cells and sub-mucosal glands, however in either NC or NLFS subjects, only epithelial cell luminal surfaces stained. ICAM-1 expression on alveolar pneumocytes (mainly type II) was slightly increased in CAL and NLFS (*p* < 0.01). Pack-years of smoking correlated with ICAM-1 expression (*r* = 0.49; *p* < 0.03).

**Conclusion:**

Airway ICAM-1 expression is markedly upregulated in CAL group, which could be crucial in rhinoviral and NTHi infections. The parenchymal ICAM-1 is affected by smoking, with no further enhancement in CAL subjects.

**Electronic supplementary material:**

The online version of this article (doi:10.1186/s12931-016-0483-8) contains supplementary material, which is available to authorized users.

## Background

Chronic obstructive pulmonary disease (COPD) is the third leading cause of mortality worldwide. It is a disabling condition resulting from damage inflicted by noxious particles and gases, mainly from cigarette smoke leading to airway remodeling and poorly-reversible airflow obstruction [1]. COPD patients are often prone to episodes of acute exacerbations of COPD (AECOPD), which drive the disease and are associated with higher mortality risks, decreased quality of life, accelerated loss of lung function and enormous health care costs [2].

COPD is seriously complicated by bacterial and viral infections. Bacteria, viruses and co-infection with both, have been shown to be important in precipitating AECOPD, with viruses being detected in 40 to 60% in PCR-based studies [3]. Viral infections are also associated with more severe exacerbations [4]. Human rhinoviruses (HRVs) make up approximately 50% of all viruses isolated from COPD patients [5]. The viral load at AECOPD is significantly higher than in the stable state [6]. The sputum viral load correlates with sputum neutrophilia and interleukin-8 levels [7], i.e., the activation of innate inflammation.

Respiratory tract epithelium is the primary target for viral pathogens. Attachment of most HRV serotypes to bronchial and alveolar airway epithelial cells is mediated by intercellular adhesion molecule 1 (ICAM-1; CD54), in more than 60%, and is essential for host-cell entry, while low-density-lipoprotein receptor and related molecules are receptors for only 10% of HRV serotypes [8]. ICAM-1 is a member of the immunoglobulin (Ig) superfamily that contains five Ig-like domains, a transmembrane domain, and a short cytoplasmic tail [9]; it is expressed constitutively on a wide variety of cells (including respiratory epithelial cells), but generally at a low basal level [10], and further inducible by the inflammatory mediators [11]. Physiologically, ICAM-1 plays a key role in stabilizing cell-cell interactions and it also facilitates leukocyte per-endothelial transmigration from blood into inflamed tissues [12].

Exposure of small-airway epithelial cells from physiologically normal smokers to cigarette smoke causes increased cell ICAM-1 expression [13]. Clinical studies have demonstrated elevated level of serum (soluble) ICAM-1 in COPD-smokers compared to non-COPD active smokers [14]. HRV itself up-regulates membrane-bound ICAM-1 expression via a NF-κβ-dependent mechanism [15]. However, the expression of ICAM-1 has not been directly investigated in large or small airways in COPD patients, which could be crucial for understanding their susceptibility to viral infections and the natural history of COPD. Anti-ICAM-1 antibody has been shown to inhibit major group HRV replication in vitro, as well as HRV-induced inflammation and lung virus RNA levels in a mice model [16].

Although most attention has been on HRV, ICAM-1 may serve as an adhesion molecule for *Haemophilus influenzae* (via bacterial P5 fimbriae), which is the main bacterial pathogen in COPD [17]. Thus, ICAM-1 is an attractive target to block not only virus-receptor binding, but also to check ICAM-1-mediated NTHi adhesion to respiratory cells.

In the present study, we have taken a new direction for human work, and test the potential for clinical relevance of some of these previous observations. We have investigated whether ICAM-1 expression is upregulated in the epithelium of airways, and alveoli, in both “normal” smokers and in patients with airflow obstruction.

## Methods

### Subjects

This study has a cross-sectional analysis of data. A total of 37 patients provided lung tissue at surgery. All had primary non-small cell lung cancer (NSCLC), with an approximately equal distribution of squamous and adenocarcinoma. Patients were classified as current smokers or ex-smokers (at least 12 months of smoking cessation). Nineteen of these had demonstrated GOLD stage I/II COPD on post-bronchodilator spirometry (FER < 70%), and ten patients had small airway disease (SAD) only, based on scalloping of the expiratory limb of the flow-volume curve and FEF_25-75_ < 70% predicted. In addition, there were eight individuals who were current smokers with no evidence of airflow obstruction, and hence designated as smokers with normal lung function (NLFS). Because of the relatively small numbers, and because of no obvious difference between them in ICAM-1 expression, the small airway disease (SAD) and definite COPD groups were merged as a single chronic airflow limitation (CAL) group. Those with a history of other chronic respiratory disorders were excluded (Table 1), including anyone with a history or clinical/physiological suggestion of asthma.

**Table 1:** Demographic and lung function data for participants

Study groups	NC		NLFS	CAL
	LA biopsy	SA resected tissue		
*n*	9	9	8	29
Male/female	4/5	6/3	3/5	13/16
Age (years)	65 (52–72)	52 (42–63)	72 (52–79)	66 (42–85)
Smoking history (pack years)	N/A	N/A	23 (0.3–60)	32.5 (0–72)
FEV_1_/FVC (%)^a^	N/A	N/A	79.5 (70–90)	68 (54.9–78)
FEF_25–75%_ (L/sec)^a^	N/A	N/A	81.5 (70–116)	41.5 (20–69)

Data expressed as median and range

*CAL* chronic airflow limitation, *FEV*_*1*_ forced expiratory volume in 1 s, *FVC* forced vital capacity, *FEF*_*25–75%*_ forced expiratory flow at 25–75%, *LA* large airway, *NC* normal control, *NLFS* normal lung function smoker, *N/A* not any, *SA* small airway

^a^Post bronchodilator values after 400 μg of salbutamol

Resected lung sections from nine non-smoking, non-COPD subjects were included as a control group (NC) for comparison of ICAM-1 expression in the small airways. Large airway biopsies (*n* = 8) from our tissue biobank were used as normal controls for the large airway resected tissue.

### Tissue section acquisition and processing

Surgical resection material well away from the main tumour, and containing non-cancer affected small (<2 mm internal diameter) and large airways, were fixed in formalin within minutes of surgery. At processing, tissue blocks were embedded in paraffin for sectioning, staining and further analyses, as previously described [18].

### Immunostaining

Sections were cut at 3 μm intervals from individual paraffin embedded blocks, stained first with hematoxylin and eosin for morphological assessment for quality and lack of damage. Following removal of paraffin and rehydration, immunostaining for ICAM-1 was done using an anti-ICAM-1 monoclonal antibody (Merck Millipore Corporation, Merck KGaA, Darmstadt, Germany, Catalogue No MAB2130, 1/250 dilution for 90 min at 20 °C, post heat retrieval). Appropriate negative and positive controls were included in the study, as previously outlined [18]. To specifically co-localize Goblet Cells and ICAM-1 staining serial sections from the same blocks were taken; immunostaining for ICAM-1 performed as above on one, and for the Goblet Cells a standard Periodic acid (May & Baker, Dagenham, England) and Schiff regent (Merck KGaA, Darmstadt, Germany) protocol with haematoxylin as a nuclear stain was used.

### Quantification of tissue sections

Computer-assisted image analysis was performed with a Leica DM 2500 microscope (Leica Microsystems, Wetzlar, Germany), Leica DFC495 camera (Leica Microsystems, Wetzlar, Germany), and Image Pro Plus 7.0 (Media Cybernetics, Inc., Rockville, MD, USA) software. An operator blinded to smoking and clinical status assessed expression of ICAM-1 on randomized and coded slides. For the epithelial analysis in both the large and small airways, we randomly chose eight fields, all without a tumor interface. ICAM-1 expression in both the large and small airway epithelium was expressed as the percentage of ICAM-1-expressing cells out of the total cells. Moreover, ICAM-1 expression was differentiated between being basal or global. An additional quantification of ICAM-1 expression in the goblet cells and sub mucosal glands was done in large airways only, with staining intensity evaluated as: 0, negative; 1, weak; 2, moderate for <20% of cells; 3, moderate for >20% of cells; 4, strong for >20% of cells. ICAM-1-expressing cells in airway reticular basement membrane (Rbm) were also quantified and normalized over length of Rbm. For alveolar ICAM-1 expression, the number of type I and type II pneumocytes expressing the antigen were quantified as percent of total alveolar epithelial cells. The length of alveolar wall quantitated was approximately 6,500 μm, which was equivalent to around 50 cells/mm of alveolar wall [19].

### Cell culture and qPCR

Bronchial epithelial cells from a commercial cell line (BEAS-2B) (CellBank, Australia) were cultured as described previously [20]. At >80% confluency, cells were stimulated with cigarette smoke extract (1%) for 4 h. RNA was isolated from cells using the using the ReliaprepTM Mini RNA cell Miniprep system (Promega, Australia). Complementary DNA (cDNA) was then generated and collected using the Promega cDNA synthesis kit (Promega, Australia). The level of ICAM-1 (H_ICAM1_1, Sigma Aldrich, USA) transcript was determined by qPCR using the Corbett Rotor-Gene 6000 system (Qiagen, Germany). Thermocycling controls were run as previously described [21]. The relative change of expression was normalized to three-reference genes (18S rRNA, β-actin, β2-microglobulin) using comparative analysis according to the manufacturer’s guidelines (Qiagen, Germany). Data were derived from two independent experiments, each performed in duplicate.

### Fixation and immunofluorescence

Cultured BEAS-2B cells were fixed and stained as previously described [22]. Briefly, post CSE-stimulation (or controls), cells were fixed with 4% paraformaldehyde (Sigma Aldrich, USA) for 20 min at room temperature and rinsed. Blocking was done with 1% bovine serum albumin (Sigma Aldrich, USA) and 1% Triton X-100 in PBS, the cells were rinsed with PBS and incubated with a 1/250 dilution of anti-ICAM-1 antibody (Merck Millipore Corporation, Merck KGaA, Darmstadt, Germany) in blocking buffer overnight at 4 °C and then incubated with a 1/500 dilution of AlexaFluor 498-conjugated goat anti-mouse secondary antibody (Molecular Probes, USA) in blocking buffer for 1 h at room temperature. The cells were rinsed and then stained with 4′, 6- diamidino-2-phenylindole (DAPI; Life Technologies, USA), diluted 1:5000 in PBS, and then incubated in the dark at room temperature for 15 min. The cells were washed three times with PBS before slides were mounted with Fluorescent-mounting Media (Dako, Australia).

Micrographs were analysed using an Olympus BX50 Fluorescence Microscope (Olympus; Tokyo, Japan) with NIH elements microscopy software (Nikon; Tokyo, Japan) and CoolSnap Hq2 CCD camera (Photometrics, USA). Image merging was completed using Adobe Photoshop C56 software (Adobe Systems, California, USA). The percentage area of ICAM-1 cell expression (green staining) was normalized by cell nuclei area (blue staining), as neither nuclear nor cell area are likely to have changed. This was measured using area of interest using the cell-tracing capacity of our computer-aided image analysis software (Image Pro Plus 7.0, Media Cybernetics, Inc., USA), using a method previously described [23].

### Statistical analysis

The distributions of these cross-sectional data were generally skewed in an upward direction, so results are presented as medians and ranges; non- parametric analyses of variance was performed first (Kruskal-Wallis Test comparing medians across all the groups of interest) and specific group differences were then explored as appropriate according to prior hypotheses (CAL verses controls) using the Mann–Whitney U test. We also performed regression analysis for ICAM-1 expression against age, FEV1, and smoking history in both the normal smoker controls and CAL groups separately. Statistical analyses were performed using GraphPad Prism 6.0 (2012) for Windows, (GraphPad Software Inc., La Jolla, CA, USA), with a two-tailed p-value ≤0.05 being considered statistically significant.

## Results

### ICAM-1 expression in epithelium of large and small airways

**Fig. 1:**
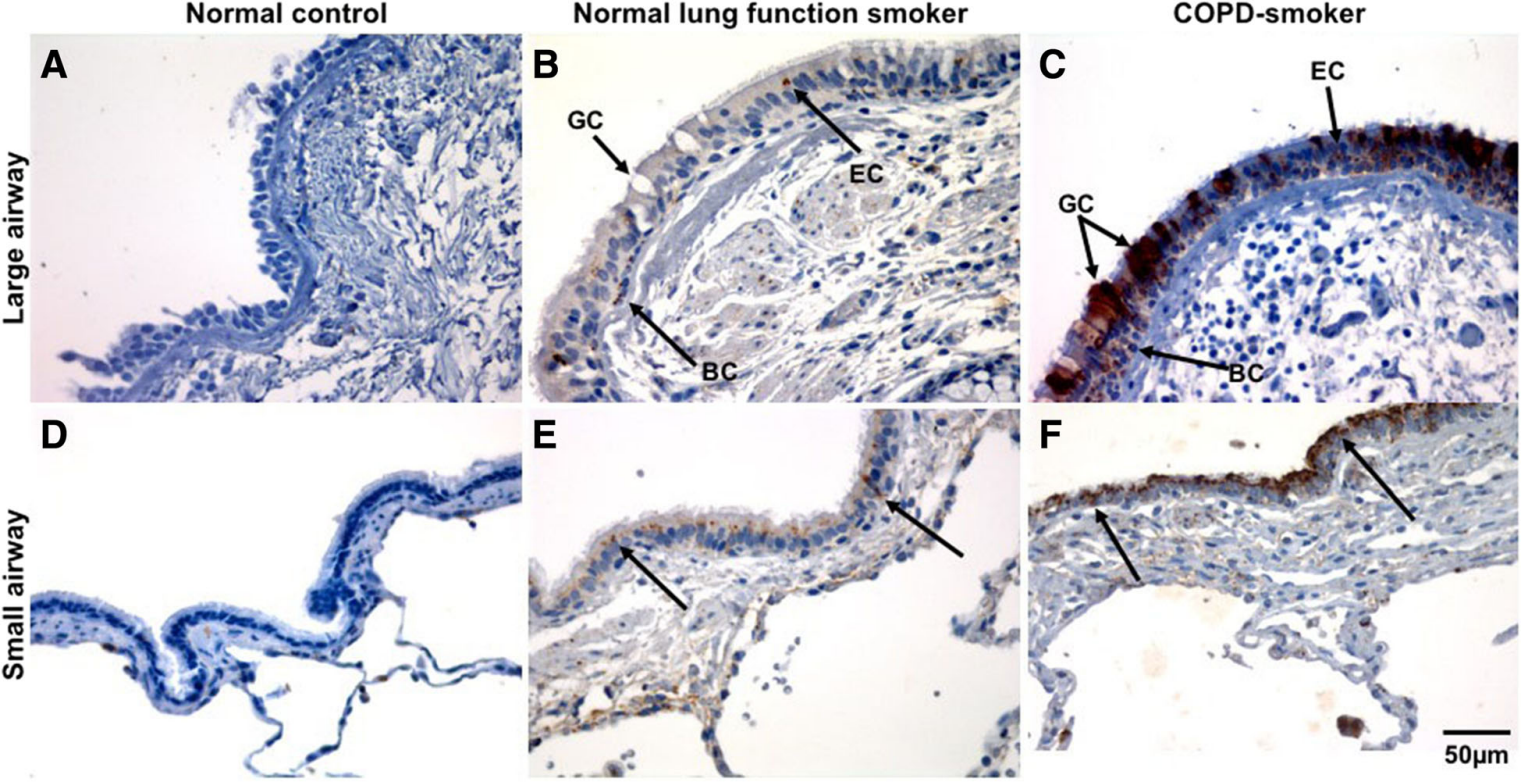
Intercellular adhesion molecule-1 (ICAM-1) expression in epithelium of large (**a**-**c**) and small airways (**d**-**f**). **a**, **d** Representative section of small airway from a never smoker showing negligible ICAM-1 staining. **b**, **e** Typical normal lung function smoker showing positive staining (showed by *black arrow*). **c**, **f** Typical COPD-smoker showing extensive ICAM-1 staining (*black arrow*). Magnification = x400. BC: basal cells; EC: epithelial cells; GC; goblet cells

**Fig. 2:**
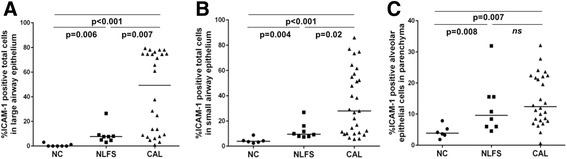
Quantification of ICAM-1-expressing cells in cross-sectional study (**a**) large airway epithelium. **b** small airway epithelium. **c** lung alveolar epithelial cells. Abbreviations: NC, normal control; CAL, chronic airflow limitation; ICAM-1, intercellular adhesion molecule-1; NLFS, normal lung-function smoker

**Fig. 3:**
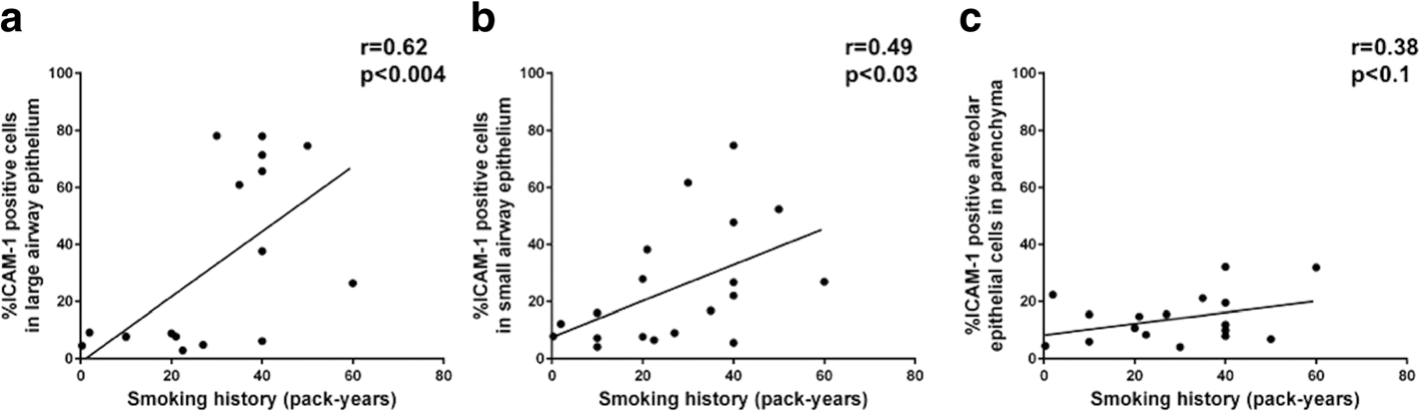
Correlation between total epithelial cells positive for ICAM-1 with smoking history (pack years). **a** large airway epithelium. **b** small airway epithelium. **c** lung alveolar epithelial cells. Abbreviations: ICAM-1, intercellular adhesion molecule-1

Compared to normal controls, epithelial staining was increased in the apical areas in the NLFS group (large airways: *p* < 0.006; small airways: *p* < 0.004), whereas in the CAL group, heavy ICAM-1 expression was observed throughout the airway epithelium (large airways: *p* < 0.001; small airways: *p* < 0.001), including both the apical and basal cells, though basal cell staining was heaviest (Figs. 1 and 2). CAL airways also had significantly greater expression than the NLFS group (large airways: *p* < 0.007; small airways: *p* < 0.02). For all current smoker groups analyzed separately, there were positive relationships between pack-year smoking history and ICAM-1 expression, for both the large and small airways (*r* = 0.50; *p* < 0.03) (Fig. 3).

### ICAM-1 positive cells in the airway reticular basement membrane (Rbm)

The Rbm in smokers and especially in COPD have been reported as hyper-cellular [24], and this was true in this study also. However, the only significant increase in the number of ICAM-1 expressing cells in the Rbm was observed in the small airway walls of the CAL group (*p* < 0.02), in cells with a fibroblast-like phenotype (Additional file 1: Figure S1).

### ICAM-1 expression in the goblet cells and sub-mucosal glands in the large airways

**Fig. 4:**
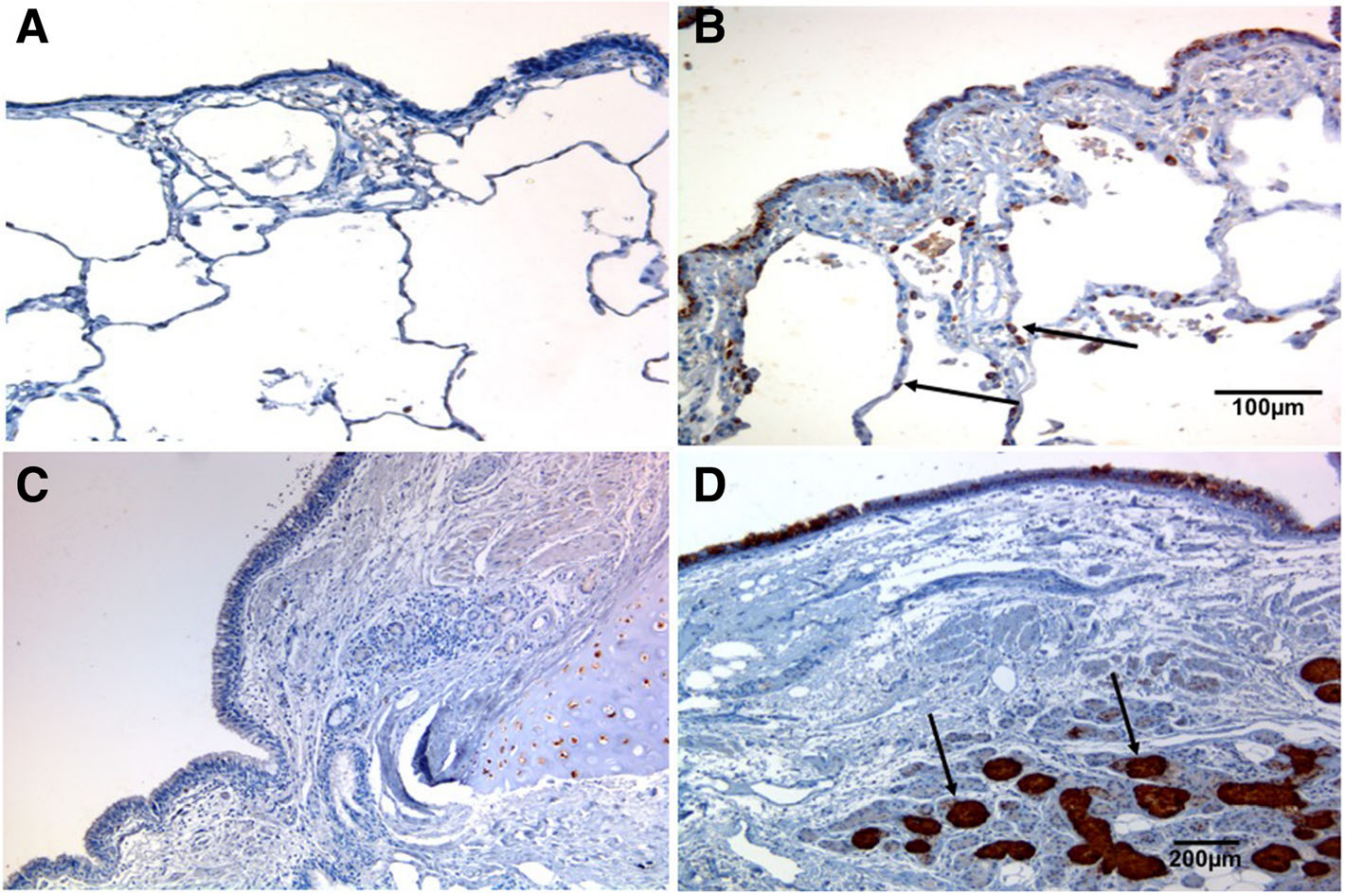
Photomicrograph showing ICAM-1 expression. (**a**-**b**) lung alveolar epithelium. (**c**-**d**) submucosal glands. (**a**) Representative sections from a never smoker. (**b**) Typical COPD-smoker showing positive staining in alveolar epithelial cells (mainly type II). (**c**) normal lung function smoker. (**d**) COPD-smoker showing extensive ICAM-1 staining in submucosal glands. Magnification: A-B=x100; C-D=x200

**Fig. 5:**
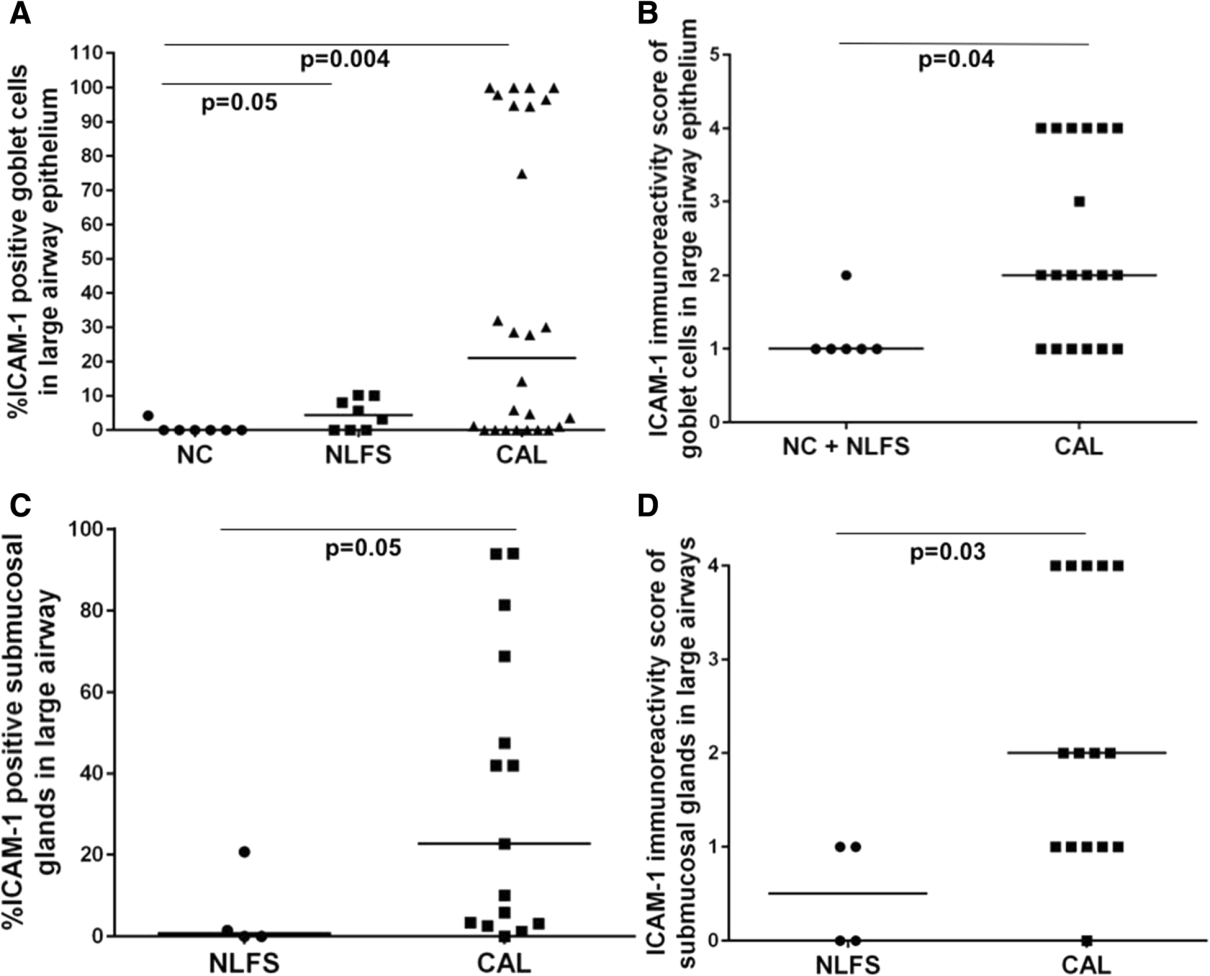
Quantification of ICAM-1-expressing goblet cells (**a**-**b**) and submucosal glands (**c**-**d**) ﻿in the cross-sectional study. (**a**) ICAM-1 expressing goblet cells in large airway epithelium. (**b**) Intensity of ICAM-1 staining of goblet cells in large airway epithelium. Only ICAM-1 positive goblet cells are included in this comparison. (**c**) ICAM-1 expressing submucosal glands. (**d**) Intensity of ICAM-1 staining of submucosal glands. Abbreviations: NC, normal control; CAL, chronic airflow limitation; ICAM-1, intercellular adhesion molecule-1; NLFS, normal lung-function smoker

**Fig. 6:**
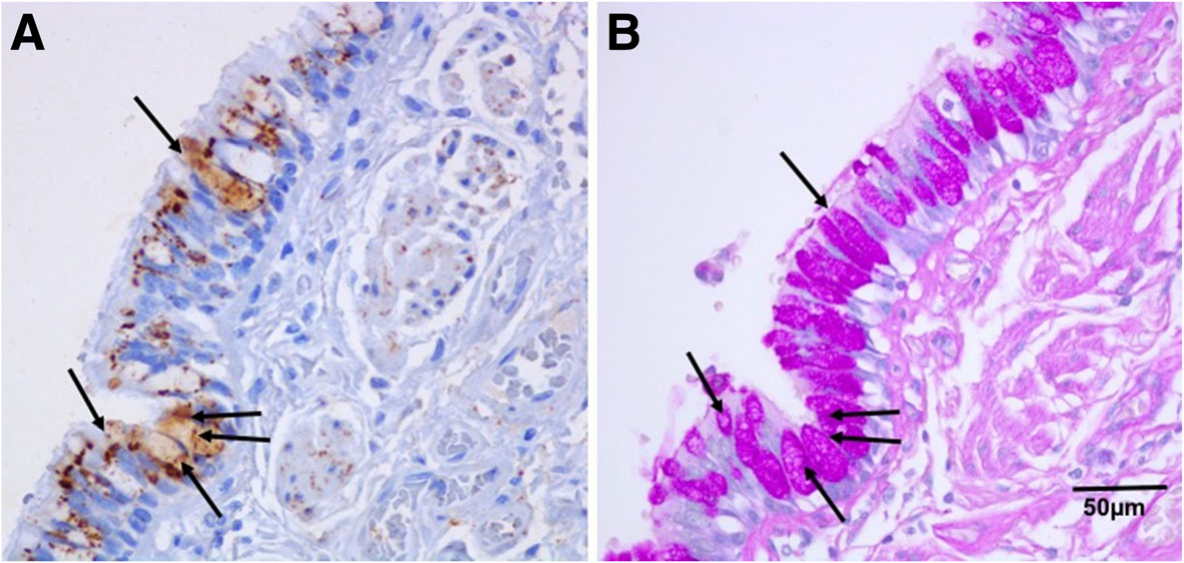
Resected lung tissue sections from a COPD-smoker showing goblet cells in of large airway epithelium. (a) ICAM-1 expression, stained with anti-ICAM-1 antibody (brown). (b) goblet cell marker, stained with Periodic Acid-Schiff (purple). Block arrows showing goblet cells expressing both ICAM-1 and PAS staining. Magnification=400x

A novel finding that we did not expect, was that Goblet Cells in the large airways of NLFS (*p* < 0.05) and CAL (*p* < 0.004) patients seemed to show especially intense ICAM-1 expression compared to control tissues (Figs. 1, 4 and 5). That these cells were indeed Goblet Cells was confirmed by the differential staining in serial sections (Fig. 6). In addition, the Goblet Cell staining intensity was significantly higher for the CAL group (*p* < 0.04), compared with NLFS and NC groups combined. Similarly, we observed increased ICAM-1 positivity in the submucosal glands in tissues from the CAL group (*p* < 0.05) compared with NLFS group, and there was also higher staining intensity (*p* < 0.03) (Figs. 4 and 5).

### ICAM-1 expression in the alveolar cells in lung parenchymal

Overall, approximately 15–30% of total epithelial cells, most frequently but not exclusive type II cells, were found to be positive in both the NLFS (*p* < 0.008) and CAL (*p* < 0.007) groups, compared with normal tissue (Figs. 2 and 4).

### Cigarette smoke extract (CSE) treatment upregulates ICAM-1 expression in bronchial epithelial cells

**Fig. 7:**
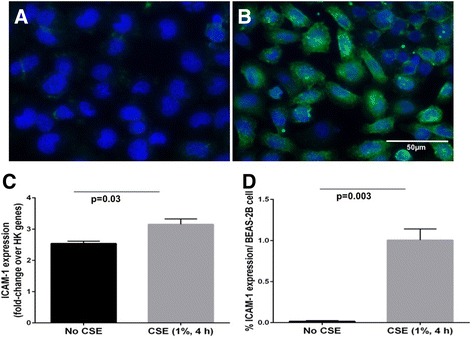
Photomicrograph showing ICAM-1 expression in bronchial epithelial cells. **a** BEAS-2B control cells. **b** BEAS-2B cells pretreated with CSE (1%, 4 h). **c** CSE 1% increases ICAM-1 transcript level, assessed by quantitative RT-PCR and normalized for three housekeeping genes. **d** CSE (1%) also increases protein expression on epithelial cell culture, quantified by computerized image analysis software. Data is presented as mean ± SEM. Magnification = 400x. (*Blue*: nuclear stain DAPI; *Green*: Alexa Fluor 488 showing ICAM-1 stain)

Limited ICAM-1 expression was observed in control BEAS-2B cells, although on a few cells only and at very low levels (Fig. 7a). CSE exposure significantly increased ICAM-1 protein expression per cell compared to untreated cells (Fig. 7b, d). Further, ICAM-1 mRNA expression (relative to three housekeeping genes) wasincreased in BEAS-2B cells exposed to CSE (*p* < 0.03; *n* = 4) compared to control cells (Fig. 7c).

## Discussion

This is the first comprehensive report of increased ICAM-1 protein expression in epithelium of both the large and small airways in smokers but especially in patients with chronic airflow limitation. This group consisted both of frank COPD plus individuals with small airway obstruction only, but we combined them because their data were very similar. There was some up-regulation in the alveolar epithelium, but this was less marked than in the airways, and uniform between smokers and all CAL groups. We also found increased ICAM-1 expression in goblet cells in large airway epithelium from smokers and CAL, but more marked in CAL. Moreover, ICAM-1 expression, both at the mRNA and protein level, was upregulated in cultured bronchial epithelial cells exposed to cigarette smoke extract. These findings, taken as a whole, may be crucial for understanding the vulnerability of smokers and especially patients with airflow obstruction to airway infections, specifically with HRV and NTHi, although for the latter, platelet-activating factor receptor (PAFr) upregulation may be of even greater importance [18, 19].

Clinical relevance of increased ICAM-1 expression in the pathogenesis of smoking-related airway diseases including COPD has been suggested previously, but mainly through indirect data, as discussed in introduction [13, 25]. Moreover, higher ICAM-1 protein expression was reported in the basal cells of bronchial epithelium from individuals with “bronchitis”, compared to normal individuals [26], but sputum concentrations of sICAM-1 did not significantly correlate with FEV_1_ [27]. Systemically, serum-sICAM-1 was higher in COPD patients than either non-smoking healthy subjects or smokers without COPD [28]. Additionally, higher concentrations of serum sICAM-1 in COPD did relate with worsening spirometry [29]. However, in contrast, Noguera et al. showed lower serum levels of sICAM-1 in patients with stable COPD than in healthy non-smokers [30]. In our study, we did not find any correlation between cellular ICAM-1-expression in the airway and either age or lung function in the CAL group, but ICAM-1 expression in both the large and small airways was significantly correlated with smoking history, with a wide range of pack-years represented.

HRV has been detected in lower airway specimens such as sputum from children with wheezy bronchitis [31], and brushed cells from allergic volunteers experimentally infected with RV16 [32] by RT-PCR and culture. Moreover, compared with normal control, cultured airway epithelial cells from patients with COPD showed increased susceptibility to RV infection, and also higher levels of mRNAs encoding ICAM-1 [33]. In normal primary human bronchial epithelial cell cultures, HRV itself upregulated membrane-bound ICAM-1 expression via NF-κβ-dependent mechanisms [15], suggesting a potential vicious cycle.

Interestingly, cultured epithelial basal cells were found to be more susceptible to RV infection than supra-basal cells, and basal cells also stained more for ICAM-1 expression [34]. The potential clinical significance of ICAM-1 as a therapeutic target has been shown by blocking the ICAM-1 receptor with anti-ICAM-1 monoclonal antibodies (MAb) in an in vitro cell-culture model [35]. In addition, corticosteroid pretreatment resulted in inhibition of HRV-induced ICAM-1 upregulation in both primary bronchial epithelial and A549 cells [36]. and one could speculate that this might be one means by which corticosteroid therapy decreases AECOPD [37].

Although respiratory tract ciliated cells are thought to be the major target for microbial pathogens, large airway goblet cells, an integral part of respiratory epithelium, and submucosal glandular cells, may also be involved. Empirically, airway viral infection results in mucus hypersecretion, which may play a role in the pathogenesis of severe airway obstruction in AECOPD. Notably, we showed increased ICAM-1 expression (both in number and intensity) in goblet cells and submucosal glands in the large airway of smokers, but especially in CAL patients, which was further confirmed by staining serially-sectioned airway wall-containing lung tissues with a specific Goblet Cell marker, Periodic acid-Schiff (PAS). Previous research has shown that HRV infection could upregulate ICAM-1-mRNA and inflammatory cytokines in submucosal gland cells, and, an anti-ICAM-1 antibody blocked both infection and production of these cytokines [38]. Thus, airway goblet cells and submucosal glands may be important potential targets of HRV induced mucus hypersecretion via viral-epithelial interactions [39], and given that there is marked hypertrophy of this glandular tissue in COPD, it again adds to the vulnerability of these patients towards HRV infections.

ICAM-1 may also serve as an adhesion receptor for NTHi [40]. Blocking cell surface ICAM-1 with specific antibody significantly reduced the adhesion of NTHi to epithelial cells [22]. It has been shown that NTHi itself upregulates ICAM-1 expression and HRV adherence [41, 42]. These studies did not take into account the possibility of co-regulation of ICAM-1 with Platelet Activating Factor receptor (PAFr), which we have previously suggested to be the main airway adhesion site for pathogenic *Haemophilus* [19], with a tight correlation between PAFr expression and NTHi adhesion to airway epithelial cells [23]. Work on potential reinforcing interactions between these two adhesion systems is now urgently needed, since novel non-antibiotic, broad anti-infective therapeutic strategies could emerge.

Alveolar epithelial cell ICAM-1 expression was increased equivalently in smokers and the CAL group, with type II cells being the predominant cell type affected. Empirically, staining was much less marked than in the airways. Burns et al. also previously reported increased ICAM-1 expression in type II pneumocytes in mice lung tissue exposed to *S. pneumoniae* [43], emphasized the possibility of ICAM-1 upregulation increasing neutrophilia, but not the possibility of increased microbial vulnerability.

The strengths of the present study include the use of abundant and relevant human tissue in well phenotyped individuals with mild-to-moderate obstructive airway disease, focusing on pathogenic mechanisms in relatively early disease with few confounding factors such as chronic bacterial infection or emphysema. We had robust numbers to give sufficient power to detect these findings, and this was confirmed by the strong statistical outcomes.

There are also a few limitations. Firstly, the study was cross-sectional and longitudinal studies of ICAM-1 expression are needed. Secondly, our control subjects were somewhat younger on average, but ages over-lapped substantially between groups and there was no suggestion of a relationship between ICAM-1 expression and age. Finally, we did not investigate viral adherence to in relation to ICAM-1 expression.

## Conclusions

In conclusion, epithelial ICAM-1 expression is upregulated throughout the respiratory tract in smokers, but is especially marked in the airway epithelium in subjects with chronic airflow obstruction, even when mild. ICAM-1 expression in Goblet Cells and sub-mucosal glands in the airway wall is also markedly increased*.* There is also an increase in the alveolar epithelium, especially in Type-2 cells, but this is a smoking effect only, and not further enhanced in COPD. Increased expression of ICAM-1 in the respiratory tract, and mostly so in the airways, could be a crucial risk factor for infection here with the most common “respiratory” viral and bacterial pathogens, and indeed such changes in pathogen adhesion sites may underlie this vulnerability of smokers and people with COPD to these specific infections which is otherwise unexplained. Translational research in this area is still in its infancy but has huge potential to provide new therapeutic targets to modify clinical management of smoking-related airflow limitation. Thus, further clinical research on anti-ICAM-1 therapies and therapies against other up-regulated microbial adhesion sites is now warranted, and indeed urgently needed.

## Additional file

Additional file 1: Figure S1. ICAM-1-positive cells in reticular basement membrane (Rbm) in the cross-sectional study. (A) large airway; (B) small airway. Abbreviations: CAL: chronic airflow limitation; NC, normal control; NLFS, normal lung-function smoker. (TIFF 924 kb)
