# Supplementary figures and images for: The main rhinovirus respiratory tract adhesion site (ICAM-1) is upregulated in smokers and patients with chronic airflow limitation (CAL)
Source: Respir Res. 2017 Jan 5;18:6. doi: 10.1186/s12931-016-0483-8 (PMC5217320; doi:10.1186/s12931-016-0483-8)

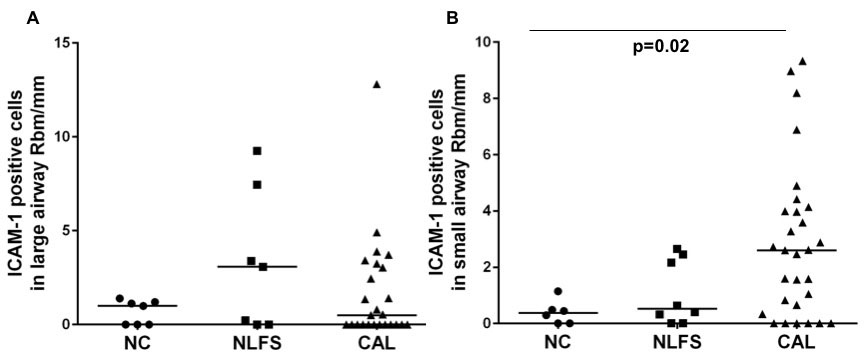

Supplement: Additional file 1: Figure S1. — ICAM-1-positive cells in reticular basement membrane (Rbm) in the cross-sectional study. (A) large airway; (B) small airway. Abbreviations: CAL: chronic airflow limitation; NC, normal control; NLFS, normal lung-function smoker. (TIFF 924 kb) [file 12931_2016_483_MOESM1_ESM.tiff]
